# Bioinformatics analysis combined with clinical sample screening reveals that leptin may be a biomarker of preeclampsia

**DOI:** 10.3389/fphys.2022.1031950

**Published:** 2023-01-04

**Authors:** Yajuan Wang, Xuening Bai, Xin Guo, Xiaoli Gao, Yuanyuan Chen, Huanrong Li, Wenjun Fan, Cha Han

**Affiliations:** ^1^ Department of Gynecology and Obstetrics, Tianjin Medical University General Hospital, Tianjin, China; ^2^ Tianjin Key Laboratory of Female Reproductive Health and Eugenics, Tianjin Medical University General Hospital, Tianjin, China

**Keywords:** leptin, preeclampsia, immune infiltration, bioinformatics, competing endogenous RNA, N 6-methyladenosine

## Abstract

**Introduction:** Preeclampsia (PE) is a gestational hypertensive disease with unclear pathogenesis. This study aimed to identify the genes that play an important role in determining the pathogenesis of PE using bioinformatics analysis and fundamental researches.

**Materials and methods:** Datasets from the Gene Expression Omnibus (GEO) database were used to screen for differentially expressed genes (DEGs). The NCBI, SangerBox, and other databases were used to analyze the functions of the DEGs. Targetscan7, miRWalk, ENCORI, DIANA TOOLS, CircBank databases, and the Cytoscape tool were used to construct the lncRNA/circRNA-miRNA- *LEP* network. SRAMP, RPISeq, RBPsuite, and catRPAID were used to analyze the RNA modifications of *LEP*. Immune cell infiltration was analyzed using the dataset GSE75010. Placental tissues from normal pregnant women and PE patients were collected, screened for gene expression using reverse transcription quantitative polymerase chain reaction (RT-qPCR) and western blotting. The results were further verified in HTR-8/SVneo cell line hypoxia model and PE mouse model.

**Results:** Our analyses revealed that *LEP* was significantly upregulated in eight datasets. Kyoto Encyclopedia of Genes and Genomes (KEGG) and Gene Ontology (GO) analyses indicated that *LEP* was involved in the JAK/STAT signaling pathway, angiogenesis, and placental development. Immune cell infiltration analysis showed that M1 and M2 macrophages differed between normal pregnancies and those in PE patients. A competing endogenous RNA (ceRNA) network was constructed, and proteins interacting with *LEP* were identified. RNA modification sites of *LEP* were also identified. Finally, the overexpression of *LEP* in PE was confirmed in clinical samples, HTR-8/SVneo cell line and PE mouse model.

**Conclusion:** Our results indicate that *LEP* overexpression is associated with PE and may be a potential diagnostic marker and therapeutic target.

## 1 Introduction

Preeclampsia (PE) is a hypertensive disease associated with pregnancy and is characterized by new-onset hypertension after 20 weeks of gestation, with or without proteinuria, which can affect multiple organs (listed, 2020). It is characterized by placental dysplasia and endothelial dysfunction (Rana et al., 2019; Han and Dong, 2021). PE is one of the leading causes of maternal, fetal, and neonatal deaths, affecting 2%–8% of pregnancies (Ives et al., 2020). PE can be divided into two subtypes: early-onset (<34 weeks) and late-onset (>34 weeks). Abnormal placental development is more strongly associated with early-onset PE, whereas late-onset PE is usually secondary to maternal microvascular disease or is associated with heredity (Dahlia Raymond and Peterson, 2011). Unfortunately, the pathogenesis of PE remains unclear, and there is no gold standard for treatment, apart from the delivery of the placenta. It is crucial to elucidate the pathogenesis of PE and identify sensitive biomarkers for predicting this disease.

Leptin (encoded by the *LEP* gene) is a polypeptide hormone secreted primarily by adipose tissue, and the placenta is also the body’s leptin producing tissue. In addition to increased maternal fat mass, placental leptin production is one of the key sources of increased maternal circulating leptin levels. Placental leptin regulates placental functions *via* autocrine or paracrine signaling, and is considered an essential signaling molecule in the reproductive system. It regulates gonadotropin production, blastocyst formation, implantation, normal placental formation, and communication between the fetus and the placenta. In addition, leptin regulates proliferation, protein synthesis, invasion, and apoptosis of placental cells, and plays a crucial role in the early stages of pregnancy (Perez-Perez et al., 2018). Nonn et al. (2021) reported elevated angiotensin IV (Ang IV) levels in the maternal circulation during pregnancy. Ang IV-induced reduction in basal mitochondrial respiration in trophoblastic cells may alter placental metabolism by increasing leptin levels. This study also suggested that the mechanisms underpinning hypertensive disease in pregnancy may be related to changes in leptin and cellular metabolism (Nonn et al., 2021). Cai et al. found that miR-519d targets *LEP* and downregulates its expression, promoting the proliferation and migration/invasion of HTR-8/SVneo cells, which may impede the development of PE (Cai et al., 2021). Huang et al. showed that miR-18b-3p was decreased and *LEP* was increased in placental tissue of PE rats. *LEP* was the direct target gene of miR-18b-3p. And human umbilical cord mesenchymal stem cells (hucMSCs) upregulated miR-18b-3p and targeted leptin, thereby reducing the levels of inflammatory factors in the placental tissues of PE rats (Huang et al., 2021). In light of these data suggesting that leptin is involved in the development of PE, this study aimed to further analyze *LEP* expression and explore additional therapeutic targets for PE.

In this study, we identified differentially expressed genes (DEGs) by comparing gene expression profiles in placental tissues from women who experienced normal pregnancy with tissues from PE patients. We then conducted Kyoto Encyclopedia of Genes and Genomes (KEGG) and Gene Ontology (GO) analyses, immune cell infiltration, and protein-protein interaction (PPI) network analyses. We also constructed a competing endogenous RNA (ceRNA) network based on the screened microRNAs (miRNAs), long non-coding RNAs (lncRNAs), and circular RNAs (circRNAs), and predicted the N^6^-methyladenosine (m6A) modification sites of *LEP* and related m6A-binding proteins. Clinical samples were collected, and HTR-8/SVneo cell line hypoxia model and PE mouse model were established, and then screened for gene expression using reverse transcription quantitative polymerase chain reaction (RT–qPCR) and western blotting. The identification and analysis of the *LEP* gene will clarify the role of *LEP* in the pathophysiology of PE and its potential association with PE, and further understand the pathogenesis of PE. This study aimed to provide more theoretical basis for clarifying that *LEP* may be a potential diagnostic marker and therapeutic target for PE.

## 2 Materials and methods

### 2.1 Data resources

All datasets [GSE10588 (Saei et al., 2021), GSE74341 (Kang et al., 2021), GSE66273 (Qi et al., 2021), GSE54618 (Saei et al., 2021), GSE4707 (Saei et al., 2021), GSE44711 (Kang et al., 2021), GSE35574 (Saei et al., 2021), and GSE24129 (Ma et al., 2021)] containing normal pregnancy and PE placental tissue sequencing data were downloaded from the GEO database (https://www.ncbi.nlm.nih.gov/). We then performed bioinformatic analyses on these data.

### 2.2 Analysis of DEG

To identify DEGs, we sorted the DEGs from all eight datasets (GSE10588, GSE74341, GSE66273, GSE54618, GSE4707, GSE44711, GSE35574, and GSE24129) in ascending order of logFC values. DEGs were selected based on *p* < 0.05. Differential expression referred to significantly altered (upregulated or downregulated) gene expression at a genomic level. An interactive Venn diagram of the upregulated genes in each dataset was prepared using Evenn (http://www.ehbio.com/test/venn/#/). Simultaneously, omicstudio (https://www.omicstudio.cn/index) was used to plot the volcano maps. Additionally, shinyGEO (https://gdancik.Shinyapps.io/shinyGEO/) was used to quantify *LEP* expression in each dataset.

#### 2.2.1 Functional annotation of *LEP*

To analyze *LEP* function in PE, NCBI (https://www.ncbi.nlm.nih.gov/) and SangerBox (http://www.sangerbox.com/) were used to perform single-gene KEGG pathway analysis and GO analysis. The Gene Ontology Biological Process (GO_BP), Gene Ontology Molecular Function (GO_MF), and Gene Ontology Cellular Component (GO_CC) terms for *LEP* were explored.

#### 2.2.2 Analysis of immune cell infiltration

The GSE75010 (Leavey et al., 2016) dataset was selected to analyze immune cell abundance in samples from 157 patients with either normal pregnancy or that with PE. For the analysis, 80 PE patients were divided into *LEP*–high and *LEP*–low expression groups, and gene set enrichment analysis (GSEA) and single sample GSEA (ssGSEA) were performed. CIBERSORT algorithm was used to determine the ratios of the immune cells. The R package “clusterProfiler” was used for GSEA analysis, and ssGSEA analysis was performed using the R package “GSVA".

#### 2.2.3 Construction of a lncRNA/circRNA-miRNA-*LEP* regulatory network

Upstream binding miRNAs of *LEP* were predicted using several target gene prediction programs, including miRWalk (http://mirwalk.umm.uni-heidelberg.de/), miRDB (http://mirdb.org/), miRabel (http://bioinfo.univ-rouen.fr/), and TargetScan7 (http://www.targetscan.org/). Only the 24 predicted miRNAs that appeared in all four programs were included in the subsequent analyses. These 24 miRNAs were also scored using TargetScan by entering the human gene symbol “*LEP*”, followed by each of the 24 miRNA names one at a time, and recording the scores that were obtained. LncRNAs targeting the screened miRNAs were predicted and analyzed using ENCORI (https://starbase.sysu.edu.cn/) and DIANA TOOLS (http://snf-515788.vm.okeanos.grnet.gr/). CircBank (http://www.circbank.cn/searchMiRNA.html) was used to predict the circRNAs. Cytoscape software was used to visualize the lncRNA/circRNA-miRNA-*LEP* regulatory network.

#### 2.2.4 Construction of a PPI network

To construct a PPI network, we performed a search on the STRING website (https://string-db.org/) using “LEP” in the “protein name” module and “*Homo sapiens*” in the organism module. We set the following key parameters: meaning of network edges (“evidence”), the minimum required interaction score [“medium confidence (0.400)”], and the maximum number of interactors to show (“no more than 20 interactors” in the 1st shell). *LEP*-binding proteins were also analyzed using GeneMANIA (https://genemania.org/) to determine the interaction between *LEP*-related proteins. The SangerBox portal was used to perform KEGG and GO analyses of *LEP*-related genes.

#### 2.2.5 RNA methylation of LEP

SRAMP (http://www.cuilab.cn/sramp/) was used to predict *LEP* m6A modification sites and their positions in the RNA secondary structure. The *LEP* FASTA mRNA sequence without introns was used for this analysis, with the parameters “Analyze RNA secondary structure” and tissue “Generic (default)". The query sequence was shown as RNA. ENCORI (https://starbase.sysu.edu.cn/ ) and RBPsuite (http://www.csbio.sjtu.edu.cn/bioinf/RBPsuite/) were used to identify proteins that can interact with *LEP*. RPISeq (http://pridb.gdcb.iastate.edu/RPISeq/) was used to predict the probability of proteins binding to *LEP*. The protein and RNA sequences were inserted in plain text format, and RF classifier and SVM classifier prediction scores were obtained. RBPsuite (http://www.csbio.sjtu.edu.cn/bioinf/RBPsuite/) and catRPAID (http://service.tartaglialab.com/page/catrapid_group) were used to identify the RNA regions of *LEP* most likely to be bound by IGF2BP3.

### 2.3 Clinical sample collection

All samples were collected from women who underwent cesarean section at the Department of Obstetrics and Gynecology of the Tianjin Medical University General Hospital between August 2021 and July 2022. This study was approved by the Medical Ethics Committee of the Tianjin Medical University General Hospital and the approval number is IRB2020-KY-008. Samples were collected with verbal informed consent from all patients. The pregnant women were divided into two groups, namely normal pregnancy and PE. The normal pregnancy group comprised pregnant women with no history of hypertension or other clinicopathological changes. Inclusion in the PE group was based on the following criteria: 1) diagnosis of systolic blood pressure of 140 mmHg or higher, or diastolic blood pressure of 90 mmHg or higher, on 2 occasions at least 4 h apart after 20 weeks of gestation; and urine protein levels of 300 mg or more every 24 h, or protein/creatinine ratio 0.3 mg/dL or higher, or test paper reading ++; or 2) in the absence of proteinuria, new-onset hypertension associated with any of the following changes: thrombocytopenia, renal insufficiency, liver function impairment, pulmonary edema, or new-onset headache unresponsive to medication that cannot be explained by other diagnoses or visual symptoms (listed, 2020). Exclusion criteria included multiple pregnancies, hypercoagulable state, gestational diabetes, chronic hypertension, autoimmune diseases, kidney and liver disease, and the use of aspirin or anticoagulants during pregnancy. Placental tissue was extracted immediately after cesarean section and placental villus tissue was dissected at 4°C. After cleaning with cold PBS, samples were quickly stored in liquid nitrogen.

### 2.4 Culture and treatment of HTR-8/SVneo cell line

The HTR-8/SVneo cell line was obtained from BeNa Culture Collection (BNCC, Beijing, China). The HTR-8/SVneo cell line was cultured with RPMI-1640 medium (Gibco BRL, Grand Island, NY, United State) supplemented with 10% fetal bovine serum (Gibco, Australia), and antibiotics (100 U/ml penicillin and 100 μg/ml streptomycin). Cells were incubated at 37°C in 5% CO_2_. For hypoxia, HTR-8/SVneo cell line was cultured in a 3-gas incubator with 1% oxygen, 5% carbon dioxide, serum-free for 24 h to simulate the process of PE.

### 2.5 Construction of the PE mouse model and sample collection

All mouse experiments were conducted in accordance with protocols approved by the Tianjin medical university animal care and use committee and followed guidelines for animal welfare. Eight-week-old C57BL/6J female and male mice were purchased from Beijing Hufukang Biotechnology Co., LTD. Mating was performed at a 2:1 ratio of male to female. We validated the overexpression of *LEP* in PE using a PE mouse model constructed by Han et al. The PE mouse model was established by injecting placenta-derived extracellular vesicles (pcEVs), which was obtained from normal pregnant mice, into pregnant C57BL/6J mice to increase circulating pcEV levels, and induced preeclampsia-like changes such as hypertension and proteinuria. In addition, two other models were used to complement this PE mouse model in this study. C57BL/6 J non-pregnant mice developed hypertension and proteinuria after injection of pcEVs to increase circulating pcEV levels. Enhanced clearance of circulating pcEVs of PE pregnant mouse model prevented clinical phenotypes of PE induced by pcEVs (Han et al., 2020). Therefore, we refer to this PE model to validate the overexpression of *LEP* in PE. In our study, on day 17–18 of gestation, pregnant mice were injected with PBS buffer through the tail vein as the control group or pcEVs as the PE group. Each pregnant mouse was injected with 100 μL. The intervention concentration (1 × 10^∧^7 pcEVs/mouse) was referenced to the concentration used by (Han et al., 2020). Blood pressure was measured 30 min after injection through the tail vein of the mice. Then, the mice were sacrificed under anesthesia, the placenta and fetus were dissected, and the collected placenta and fetus were weighed.

The detailed steps for obtaining pcEVs are as follows, placentas from normal pregnant C57BL/6J mice between 17, 18 days were washed with ice-cold sterile PBS, cut into small pieces, and frozen in liquid nitrogen. Placentas were gently added to 1 ml PBS and homogenized at 4°C. The placenta homogenates were centrifuged at 1,500 × *g* for 20 min at 4°C to remove intact cells. The supernatant was centrifuged at 13,000 × *g* at 4°C for 2 min to remove large cell debris, and then centrifuged at 4°C at 100,000 × *g* for 60 min (twice) to collect pcEVs and resuspend in PBS (Han et al., 2020).

### 2.6 RT-qPCR

Total RNA was extracted using the Trizol reagent (Thermo Fisher Scientific, Inc. United State), and cDNA was obtained by reverse transcription using the TransScript^®^ First-Strand cDNA Synthesis SuperMix (Transgen Biotech Corporation, China). The cDNA was amplified using the Hieff UNICON universal Blue qPCR SYBR Green Master Mix kit (YEASEN Corporation, China). The reaction volume was 20 μL, including 10 μL of Universal Blue qPCR SYBR Green Master Mix, 7.6 μL of nucleic acid-free water, 0.2 μL of each primer, and 2 μL of cDNA product. The PCR cycling conditions were as follows: 95°C for 2 min for 1 cycle, 95°C for 10 s, 60°C for 30 s for 40 cycles, followed by the melting curve stage. *GAPDH* was used as an internal reference and relative mRNA expression was calculated using the 2^−ΔΔCT^ method. The following primer sequences were used for amplification:

GAPDH
Forward: 5′- AAG​GTG​AAG​GTC​GGA​GTC​AAC-3′,
Reverse: 5′- GGG​GTC​ATT​GAT​GGC​AAC​AAT -3′,
LEP
Forward: 5′- TGC​CTT​CCA​GAA​ACG​TGA​TCC -3′,
Reverse: 5′- CTC​TGT​GGA​GTA​GCC​TGA​AGC -3’.
LINC00473
Forward: 5′- TCA​TTT​CCC​TAC​CTG​CTC​CT-3′,
Reverse: 5′- CAG​TGT​CTG​CAC​ATC​GCT​AAT-3’.

### 2.7 Western blot analysis

Total protein was extracted from each 50 mg placenta sample using 300 μL of protein lysis buffer, which was composed of RIPA buffer (Solarbio, China), PMSF (Solarbio, China), protease inhibitor (MedChemExpress, China), and DNA enzyme inhibitor (Solarbio, China). The total protein concentration was determined using a bicinchoninic acid (BCA) assay (Solarbio, China). Protein samples (30 μg) were electrophoresed on a 15% sodium dodecyl sulfate polyacrylamide gel at 80 V for 30 min and 120 V for 60 min. After electrophoresis, the protein samples were transferred to polyvinylidene fluoride (PVDF) membrane at 80 V, for 100 min, and the blot was washed thrice with TBST for 5 min. After blocking non-specific binding with 5% milk for 2 h at room temperature, the membranes were washed thrice with TBST for 5 min. The membranes were incubated in mouse anti-leptin (Sino Biological, China), rabbit anti-leptin (ABclonal, China) or rabbit anti-GAPDH (Cell Signaling Technology Inc. United State) and mouse anti-β actin (ZSGB-BIO, China) primary antibodies at 4 °C with low agitation overnight. The next day, the primary antibody was recovered and the membrane was washed thrice with TBST for 5 min. The blots were incubated with horseradish peroxidase (HRP)-labeled goat anti-mouse/rabbit IgG (ZSGB-BIO, China) at room temperature for 1 h, then washed thrice with TBST for 10 min. Protein expression was visualized using the chemiluminescence with GAPDH used as a loading control. The relative expression of leptin was calculated as the ratio of the optical density values of leptin to GAPDH. ImageJ was used to measure the gray values.

### 2.8 Statistical analysis

Statistical and image analyses were performed using GraphPad Prism 8.0 software (GraphPad Software, San Diego, CA, United State). Data are expressed as mean ± standard error of the mean (SEM). Comparisons between two groups were performed using an independent sample *t*-test. The criterion for statistical significance was set at *p* < 0.05.

## 3 Results

### 3.1 Identification of LEP overexpression in PE

**FIGURE 1:**
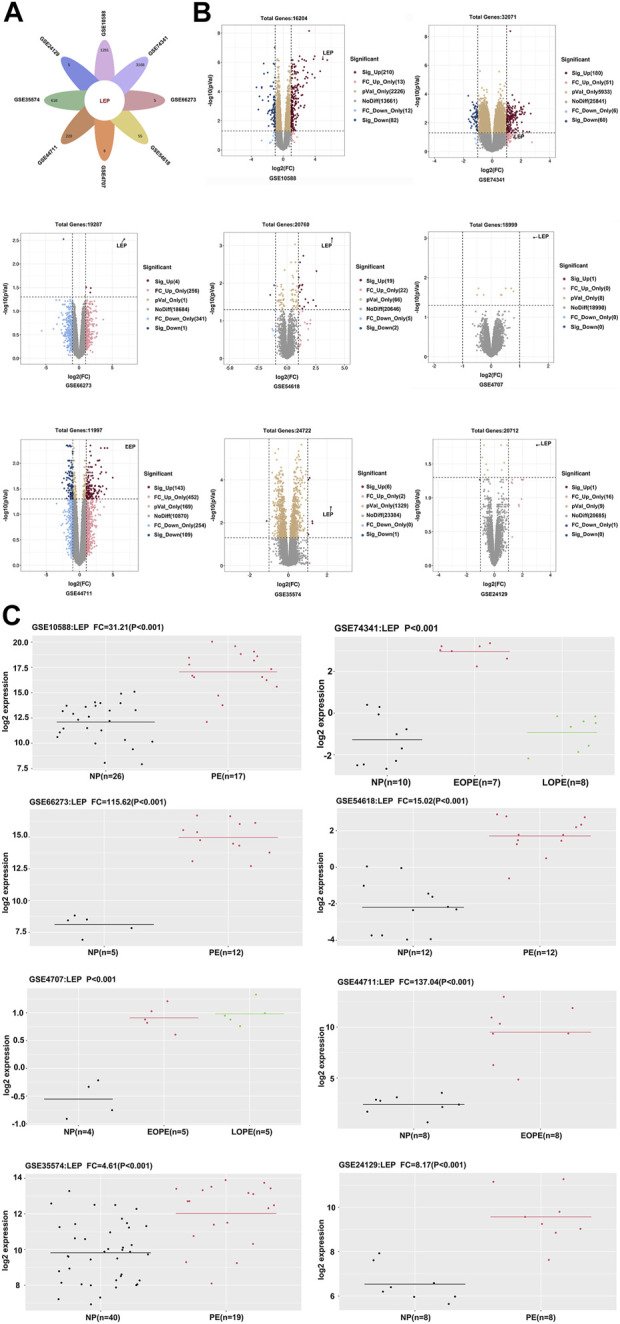
Identification of differentially expressed genes. **(A)** Venn diagram showing one upregulated gene, *LEP.* **(B)** Volcano plot of gene expression profile data in GSE10588, GSE74341, GSE66273, GSE54618, GSE4707, GSE44711, GSE35574, and GSE24129 datasets. **(C)** The quantity of *LEP* expression in the GSE10588, GSE74341, GSE66273, GSE54618, GSE4707, GSE44711, GSE35574, and GSE24129 datasets.

We selected eight datasets of PE data (GSE10588, GSE74341, GSE66273, GSE54618, GSE4707, GSE44711, GSE35574, and GSE24129) from the GEO database, based on the literature. Our analyses revealed that *LEP* was significantly overexpressed in all eight databases (Figure 1A). *LEP* expression was higher in the PE group compared with the normal pregnancy group. In datasets GSE74341 and GSE4707, PE was divided into early-onset and late-onset PE, and dataset GSE74341 showed that *LEP* expression was higher in early-onset PE than in late-onset PE (Figure 1C). And *LEP* is labeled in the volcano maps of each dataset (*p* < 0.05) (Figure 1B). Together, these results show that *LEP* is significantly upregulated in PE.

### 3.2 Functional annotation of LEP

Next, we focused on the relationship between *LEP* and PE development. Single-gene KEGG and GO analyses of *LEP* were performed to evaluate its biological functions (Tables 1,2). Single-gene KEGG pathway analysis showed that *LEP* is associated with the Janus kinase/signal transducer and activator of transcription (JAK/STAT) and adipocytokine signaling pathways, HIF-1-alpha transcription factor network, and developmental biology. The GO_BP annotation showed that *LEP* is involved in angiogenesis, placental development, response to estradiol, regulation of blood pressure, female pregnancy, positive regulation of tyrosine phosphorylation of STAT protein, positive regulation of receptor signaling pathway *via* JAK/STAT, positive regulation of MAPK cascade, and other biological processes; while GO_CC annotation revealed links to extracellular region, extracellular space, and cytoplasm. The GO_MF annotations were mainly related to peptide receptor binding and hormone activity.

**TABLE 1:** Single-gene KEGG Pathway in NCBI and SangerBox database.

Pathway	Source
Amp-activated protein kinase (ampk) signaling	WikiPathways
Adipogenesis	WikiPathways
Antipsychotics pathway (metabolic side effects), pharmacodynamics	PharmGKB
Developmental biology	Reactome
Differentiation of white and brown adipocyte	WikiPathways
HIF-1-Alpha transcription factor network	Pathway Interaction Database
Incretin synthesis, secretion, and inactivation	Reactome
Leptin and adiponectin	WikiPathways
Leptin-Insulin Signaling Overlap	WikiPathways
Metabolism of proteins	Reactome
Nonalcoholic fatty liver disease	WikiPathways
Peptide hormone metabolism	Reactome
Signaling pathways	Reactome
Signaling by leptin	Reactome
Signaling events mediated by ptp1b	Pathway Interaction Database
Spinal cord injury	WikiPathways
Synthesis, secretion, and deacylation of ghrelin	Reactome
Synthesis, secretion, and inactivation of glucagon-like peptide-1 (GLP-1)	Reactome
Transcription factor regulation in adipogenesis	WikiPathways
Transcriptional regulation of white adipocyte differentiation	Reactome
Cytokine-cytokine receptor interaction	SangerBox database
Neuroactive ligand-receptor interaction	SangerBox database
Ampk signaling pathway	SangerBox database
JAK/STAT signaling pathway	SangerBox database
ADipocytokine signaling pathway	SangerBox database
Non-alcoholic fatty liver disease (nafld)	SangerBox database

**TABLE 2:** Single-gene GO analysis in SangerBox database.

GO id	Name space	Name	Rref database
GO:0000122	Biological_process	Negative regulation of transcription by RNA polymerase II	GO_REF:0000107
GO:0001525	Biological_process	Angiogenesis	PMID:19910644
GO:0001525	Biological_process	Angiogenesis	PMID:21771332
GO:0001542	Biological_process	Ovulation from ovarian follicle	GO_REF:0000107
GO:0001666	Biological_process	Response to hypoxia	GO_REF:0000107
GO:0001890	Biological_process	Placenta development	PMID:17957153
GO:0001936	Biological_process	Regulation of endothelial cell proliferation	PMID:11460888
GO:0002021	Biological_process	Response to dietary excess	GO_REF:0000107
GO:0002021	Biological_process	Response to dietary excess	GO_REF:0000107
GO:0003300	Biological_process	Cardiac muscle hypertrophy	GO_REF:0000107
GO:0005576	Cellular_component	Extracellular region	Reactome:R-HSA-1183003
GO:0005615	Cellular_component	Extracellular space	GO_REF:0000024
GO:0005737	Cellular_component	Cytoplasm	GO_REF:0000107
GO:0006006	Biological_process	Glucose metabolic process	GO_REF:0000107
GO:0006111	Biological_process	Regulation of gluconeogenesis	GO_REF:0000107
GO:0006114	Biological_process	Glycerol biosynthetic process	GO_REF:0000107
GO:0006635	Biological_process	Fatty acid beta-oxidation	GO_REF:0000107
GO:0006909	Biological_process	Phagocytosis	GO_REF:0000107
GO:0006909	Biological_process	Phagocytosis	GO_REF:0000107
GO:0007565	Biological_process	Female pregnancy	GO_REF:0000107
GO:0007623	Biological_process	Circadian rhythm	GO_REF:0000107
GO:0008203	Biological_process	Cholesterol metabolic process	GO_REF:0000107
GO:0008206	Biological_process	Bile acid metabolic process	GO_REF:0000107
GO:0008217	Biological_process	Regulation of blood pressure	GO_REF:0,000107
GO:0008340	Biological_process	Determination of adult lifespan	GO_REF:0000107
GO:0008343	Biological_process	Adult feeding behavior	GO_REF:0000024
GO:0010507	Biological_process	Negative regulation of autophagy	PMID:25060689
GO:0010888	Biological_process	Negative regulation of lipid storage	GO_REF:0000107
GO:0014068	Biological_process	Positive regulation of phosphatidylinositol 3-kinase signaling	GO_REF:0000024
GO:0014068	Biological_process	Positive regulation of phosphatidylinositol 3-kinase signaling	GO_REF:0000024
GO:0014068	Biological_process	Positive regulation of phosphatidylinositol 3-kinase signaling	PMID:24340098
GO:0014823	Biological_process	Response to activity	GO_REF:0000107
GO:0019953	Biological_process	Sexual reproduction	PMID:8589726
GO:0021954	Biological_process	Central nervous system neuron development	GO_REF:0000107
GO:0030073	Biological_process	Insulin secretion	GO_REF:0000107
GO:0030217	Biological_process	T-cell differentiation	GO_REF:0000024
GO:0030300	Biological_process	Regulation of intestinal cholesterol absorption	GO_REF:0000107
GO:0032008	Biological_process	Positive regulation of TOR signaling	PMID:25060689
GO:0032099	Biological_process	Negative regulation of appetite	GO_REF:0000024
GO:0032310	Biological_process	Prostaglandin secretion	PMID:19688109
GO:0032355	Biological_process	Response to estradiol	GO_REF:0000107
GO:0032615	Biological_process	Interleukin-12 production	GO_REF:0000107
GO:0032760	Biological_process	Positive regulation of tumor necrosis factor production	GO_REF:0000107
GO:0032814	Biological_process	Regulation of natural killer cell activation	PMID:12504075
GO:0032817	Biological_process	Regulation of natural killer cell proliferation	PMID:12504075
GO:0033197	Biological_process	Response to vitamin E	GO_REF:0000107
GO:0033210	Biological_process	Leptin-mediated signaling pathway	GO_REF:0000024
GO:0033210	Biological_process	Leptin-mediated signaling pathway	GO_REF:0000024
GO:0033686	Biological_process	Positive regulation of luteinizing hormone secretion	GO_REF:0000107
GO:0035360	Biological_process	Positive regulation of peroxisome proliferator activated receptor signaling pathway	GO_REF:0000107
GO:0035556	Biological_process	Intracellular signal transduction	GO_REF:0000107
GO:0035630	Biological_process	Bone mineralization involved in bone maturation	GO_REF:0000107
GO:0035904	Biological_process	Aorta development	GO_REF:0000107
GO:0038108	Biological_process	Negative regulation of appetite by leptin-mediated signaling pathway	GO_REF:0000024
GO:0042102	Biological_process	Positive regulation of T-cell proliferation	PMID:25060689
GO:0042269	Biological_process	Regulation of natural killer cell mediated cytotoxicity	PMID:12504075
GO:0042307	Biological_process	Positive regulation of protein import into nucleus	GO_REF:0000107
GO:0042445	Biological_process	Hormone metabolic process	GO_REF:0000107
GO:0042531	Biological_process	Positive regulation of tyrosine phosphorylation of STAT protein	GO_REF:0000107
GO:0042593	Biological_process	Glucose homeostasis	GO_REF:0000107
GO:0042755	Biological_process	Eating behavior	GO_REF:0000107
GO:0043066	Biological_process	Negative regulation of apoptotic process	GO_REF:0000107
GO:0043270	Biological_process	Positive regulation of ion transport	GO_REF:0000107
GO:0043410	Biological_process	Positive regulation of MAPK cascade	GO_REF:0000024
GO:0043410	Biological_process	Positive regulation of MAPK cascade	PMID:24340098
GO:0044320	Biological_process	Cellular response to leptin stimulus	PMID:17344214
GO:0045471	Biological_process	Response to ethanol	GO_REF:0000107
GO:0045765	Biological_process	Regulation of angiogenesis	PMID:11460888
GO:0045906	Biological_process	Negative regulation of vasoconstriction	GO_REF:0000107
GO:0046325	Biological_process	Negative regulation of glucose import	PMID:24340098
GO:0046427	Biological_process	Positive regulation of receptor signaling pathway *via* JAK/STAT	PMID:17344214
GO:0046628	Biological_process	Positive regulation of insulin receptor signaling pathway	GO_REF:0000107
GO:0046850	Biological_process	Regulation of bone remodeling	GO_REF:0000024
GO:0046881	Biological_process	Positive regulation of follicle-stimulating hormone secretion	GO_REF:0000107
GO:0048639	Biological_process	Positive regulation of developmental growth	PMID:17957153
GO:0050796	Biological_process	Regulation of insulin secretion	GO_REF:0000107
GO:0050810	Biological_process	Regulation of steroid biosynthetic process	GO_REF:0000107
GO:0050892	Biological_process	Intestinal absorption	PMID:24340098
GO:0050901	Biological_process	Leukocyte tethering or rolling	GO_REF:0000107
GO:0050999	Biological_process	Regulation of nitric-oxide synthase activity	PMID:15899045
GO:0051541	Biological_process	Elastin metabolic process	GO_REF:0000107
GO:0051726	Biological_process	Regulation of cell cycle	PMID:17344214
GO:0051897	Biological_process	Positive regulation of protein kinase B signaling	GO_REF:0000024
GO:0060587	Biological_process	Regulation of lipoprotein lipid oxidation	GO_REF:0000107
GO:0060612	Biological_process	Adipose tissue development	GO_REF:0000107
GO:0061037	Biological_process	Negative regulation of cartilage development	GO_REF:0000107
GO:0070093	Biological_process	Negative regulation of glucagon secretion	GO_REF:0000107
GO:0071298	Biological_process	Cellular response to l-ascorbic acid	GO_REF:0000107
GO:0071300	Biological_process	Cellular response to retinoic acid	GO_REF:0000107
GO:0072604	Biological_process	Interleukin-6 secretion	PMID:1968809
GO:0072606	Biological_process	Interleukin-8 secretion	PMID:19688109
GO:0090335	Biological_process	Regulation of brown fat cell differentiation	GO_REF:0000024
GO:0098868	Biological_process	Bone growth	GO_REF:0000024
GO:0120162	Biological_process	Positive regulation of cold-induced thermogenesis	PMID:27986616
GO:1900015	Biological_process	Regulation of cytokine production involved in inflammatory response	PMID:19688109
GO:1900745	Biological_process	Positive regulation of p38MAPK cascade	PMID:24340098
GO:1904651	Biological_process	Positive regulation of fat cell apoptotic process	GO_REF:0000107
GO:1990051	Biological_process	Activation of protein kinase C activity	PMID:24340098
GO:2000379	Biological_process	Positive regulation of reactive oxygen species metabolic process	GO_REF:0000107
GO:2000486	Biological_process	Negative regulation of glutamine transport	GO_REF:0000107
GO:2000491	Biological_process	Positive regulation of hepatic stellate cell activation	GO_REF:0000107
GO:0014068	Biological_process	Positive regulation of phosphatidylinositol 3-kinase signaling	PMID:21873635
GO:0046427	Biological_process	Positive regulation of receptor signaling pathway *via* JAK/STAT	PMID:21873635
GO:1990051	Biological_process	Activation of protein kinase C activity	PMID:21873635
GO:0006112	Biological_process	Energy reserve metabolic process	PMID:21873635
GO:0051428	Molecular_function	Peptide hormone receptor binding	PMID:21873635
GO:0006629	Biological_process	Lipid metabolic process	PMID:21873635
GO:0032008	Biological_process	Positive regulation of TOR signaling	PMID:21873635
GO:0005615	Cellular_component	Extracellular space	PMID:21873635
GO:0007260	Biological_process	Tyrosine phosphorylation of STAT protein	PMID:21873635
GO:0032868	Biological_process	Response to insulin	PMID:21873635
GO:1900745	Biological_process	Positive regulation of p38 MAPK cascade	PMID:21873635
GO:0038108	Biological_process	Negative regulation of appetite by leptin-mediated signaling pathway	PMID:21873635
GO:0005179	Molecular_function	Hormone activity	PMID:21873635

### 3.3 Analysis of immune cell infiltration

**FIGURE 2:**
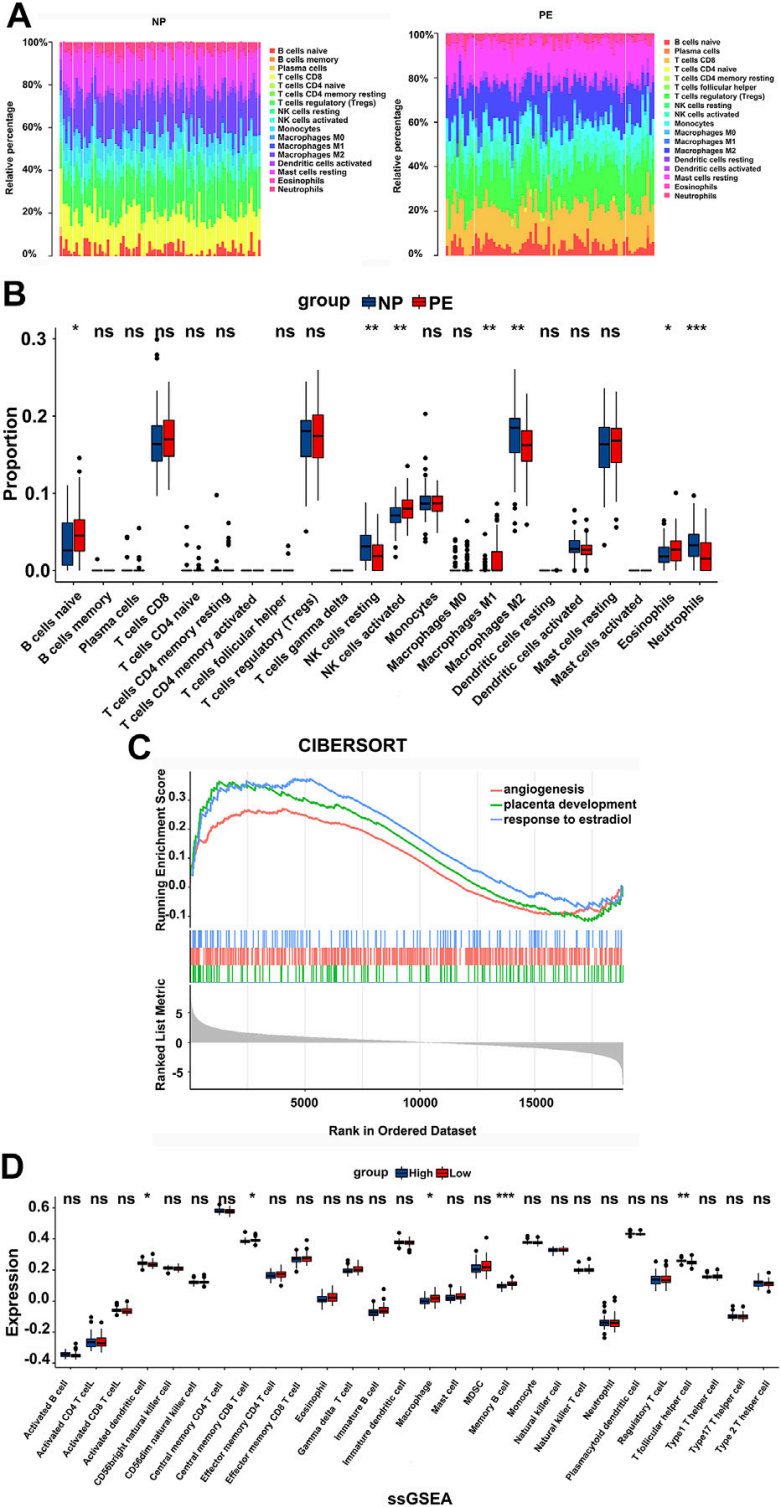
Placental immune cell infiltration analysis. **(A,B)** Differences in placental immune cell infiltration between normal pregnancy and PE. **(C)** A cohort of 80 PE patients were divided into *LEP* high and low expression groups for GSEA. Angiogenesis: NES: 1.27; FDR: 0.16; *p*-value: 0.016. Placenta development: NES: 1.51; FDR: 0.13; *p*-value: 0.007. Response to estradiol: NES: 1.54; FDR: 0.11; *p*-value: 0.005; **(D)** ssGSEA of the *LEP* high and low expression groups, to analyze immune cell infiltration. ns (not significant), * *p* < 0.05, ** *p* < 0.01, *** *p* < 0.001.

157 samples from GSE75010 were selected to study the infiltration of immune cells into normal pregnancy and PE placental tissues. Among the 22 immune cell types assessed, the number of naive B Cells, resting NK cells, activated NK cells, M1 macrophages, M2 macrophages, and eosinophils was significantly different between the two groups (Figures 2A,B). Next, 80 PE patients were divided into *LEP* high and low expression groups for GSEA. The results revealed that angiogenesis, placental development, and response to estradiol were enriched (Figure 2C). Concurrent ssGSEA revealed differences in activated dendritic cells, central memory CD8 T Cells, macrophages, memory B Cells, and T follicular helper cells between the two groups (Figure 2D).

### 3.4 Identification of differentially expressed miRNAs (DEMs)

**FIGURE 3:**
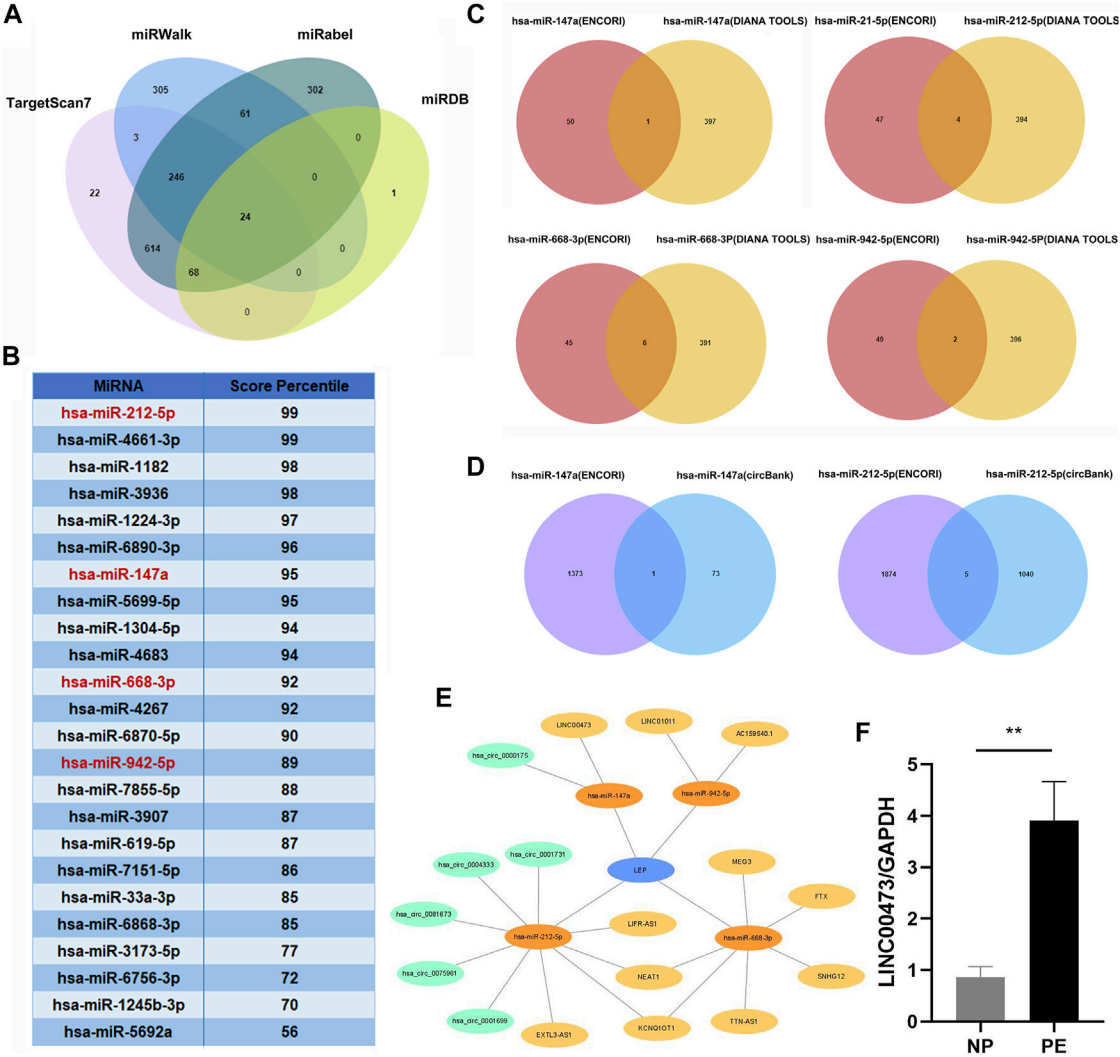
Construction of a lncRNA/circRNA-miRNA-*LEP* regulatory network. **(A)** The upstream miRNAs of *LEP* were predicted by miRWalk, miRDB, Targetscan and miRabel databases and the intersecting miRNAs were selected (24 miRNAs). **(B)** The 24 miRNAs targeting *LEP* were scored in the Targetscan database. **(C)** The upstream lncRNAs targeting miRNAs were predicted using the ENCORI and DIANA TOOLS databases and the intersecting lncRNAs were selected (11 lncRNAs). **(D)** The upstream circRNAs targeting miRNAs were predicted using the ENCORI and CircBank databases and the intersecting circRNAs were selected (6 circRNAs) **(E)** The lncRNA/circRNA-miRNA-*LEP* regulatory network was constructed using Cytoscape. **(F)** RT-qPCR analysis of LINC00473 expression in PE placental tissue samples (*n* = 10) compared with that in normal pregnancy placental tissue samples (*n* = 10). * *p* < 0.05, ** *p* < 0.01.

We screened the miRWalk, miRDB, TargetScan, and miRabel databases and identified 24 miRNAs that potentially target *LEP* mRNAs (Figure 3A). These included hsa-miR-212-5p, hsa-miR-4661-3p, hsa-miR-1182, hsa-miR-3936, hsa-miR-1224-3p, hsa-miR-6890-3p, hsa-miR-147a, hsa-miR-5699-5p, hsa-miR-1304-5p, hsa-miR-4683, hsa-miR-668-3p, hsa-miR-4267, hsa-miR-6870-5p, hsa-miR-942-5p, hsa-miR-7855-5p, hsa-miR-3907, hsa-miR-619-5p, hsa-miR-7151-5p, hsa-miR-33a-3p, hsa-miR-6868-3p, hsa-miR-3173-5p, hsa-miR-6756-3p, hsa-miR-1245b-3p, and hsa-miR-5692a. Additionally, these 24 miRNAs were scored (Figure 3B). KEGG and GO analyses were performed on the selected miRNAs to explore their functions (Tables 3,4). KEGG analysis showed significant differences in the relevance of the TGF-beta, PI3K-Akt, MAPK, and JAK/STAT signaling pathways. GO analysis revealed comparisons with the stress-activated MAPK cascade, immune system process, blood coagulation, and *in utero* embryonic development.

**TABLE 3:** miRNA KEGG analysis (DIANA TOOLS).

KEGG pathway	*p*-value	Genes	miRNAs
TGF-beta signaling pathway	1.13E-07	53	20
ErbB signaling pathway	7.46E-06	60	22
N-Glycan biosynthesis	1.53E-05	30	17
FoxO signaling pathway	1.53E-05	83	21
Mucin type O-Glycan biosynthesis	1.62E-05	18	15
Adrenergic signaling in cardiomyocytes	5.90E-05	83	23
Signaling pathways regulating pluripotency of stem cells	7.58E-05	81	21
Hippo signaling pathway	8.01E-05	92	23
Ras signaling pathway	0.000154,077	124	24
Regulation of actin cytoskeleton	0.000185,611	122	24
Rap1 signaling pathway	0.000224,072	122	23
Glutamatergic synapse	0.001,802,716	66	21
Glycosaminoglycan biosynthesis - chondroitin sulfate/dermatan sulfate	0.002,242,769	12	10
Adherens junction	0.002,242,769	48	16
Thyroid hormone signaling pathway	0.002,242,769	69	21
Long-term depression	0.003,659,698	39	21
Estrogen signaling pathway	0.005,232,062	53	20
Oxytocin signaling pathway	0.005,232,062	86	23
Axon guidance	0.00627,603	74	23
PI3K-Akt signaling pathway	0.00627,603	176	24
Sphingolipid signaling pathway	0.006,351,843	65	20
Phosphatidylinositol signaling system	0.008,158,926	43	19
Melanogenesis	0.010,385,807	57	17
Focal adhesion	0.011,193,156	111	24
MAPK signaling pathway	0.012,068,648	133	22
Bacterial invasion of epithelial cells	0.016,363,283	45	20
JAK/STAT signaling pathway	0.017,172,374	85	23
Dilated cardiomyopathy	0.020,923,949	52	21
Pantothenate and CoA biosynthesis	0.02,187,599	13	9
Dorso-ventral axis formation	0.02,187,599	19	12
Endocytosis	0.02,187,599	108	23
Morphine addiction	0.02,368,039	51	18
T-cell receptor signaling pathway	0.024,226,069	60	22
Circadian entrainment	0.026,972,795	52	21
Ubiquitin mediated proteolysis	0.031,051,668	76	23
Long-term potentiation	0.044,819,592	39	19

**TABLE 4:** miRNA GO Category (DIANA TOOLS).

GO category	*p*-value	Genes	miRNAs
Organelle	1.25E-46	325	1
Cellular nitrogen compound metabolic process	1.33E-36	195	1
Ion binding	5.61E-30	217	1
Biosynthetic process	8.70E-25	159	1
Molecular_function	7.87E-15	430	1
Nucleic acid binding transcription factor activity	3.69E-12	53	1
Transcription, DNA-templated	1.43E-07	93	1
Cytosol	1.56E-07	94	1
Catabolic process	2.39E-07	69	1
RNA binding	3.00E-07	71	1
Neurotrophin TRK receptor signaling pathway	5.46E-07	17	1
Gene expression	7.36E-07	27	1
Enzyme binding	9.39E-07	51	1
Biological_process	3.20E-06	405	1
Nucleoplasm	4.30E-06	47	1
Cell death	7.95E-06	39	1
Nucleobase-containing compound catabolic process	1.14E-05	37	1
Symbiosis, encompassing mutualism through parasitism	1.25E-05	24	1
Cellular protein metabolic process	1.31E-05	22	1
Cellular protein modification process	1.67E-05	71	1
DNA binding	2.38E-05	125	1
Cellular_component	2.38E-05	411	1
Transcription from RNA polymerase II promoter	2.95E-05	32	1
Viral process	4.64E-05	21	1
Fc-epsilon receptor signaling pathway	5.36E-05	11	1
Protein complex	8.15E-05	108	1
Response to stress	0.00011875	70	1
Toll-like receptor 10 signaling pathway	0.000148,087	7	1
Poly(A) RNA binding	0.000206,746	61	1
Toll-like receptor TLR1:TLR2 signaling pathway	0.00022013	7	1
Toll-like receptor TLR6:TLR2 signaling pathway	0.00022013	7	1
Toll-like receptor 5 signaling pathway	0.000337,865	7	1
Activation of signaling protein activity involved in unfolded protein response	0.000390,124	7	1
Virion assembly	0.000559,594	5	1
Toll-like receptor 9 signaling pathway	0.000603,341	7	1
Toll-like receptor 2 signaling pathway	0.001,511,812	7	1
Epidermal growth factor receptor signaling pathway	0.001,564,322	12	1
Stress-activated MAPK cascade	0.001,940,873	6	1
TRIF-dependent toll-like receptor signaling pathway	0.003,796,881	6	1
Toll-like receptor signaling pathway	0.004,187,716	8	1
Translation factor activity, nucleic acid binding	0.004,187,716	9	1
Small molecule metabolic process	0.004,187,716	62	1
MyD88-independent toll-like receptor signaling pathway	0.006,760,211	6	1
Viral protein processing	0.007,430,104	3	1
Mitotic cell cycle	0.007,570,654	15	1
Toll-like receptor 4 signaling pathway	0.007,635,799	7	1
MyD88-dependent toll-like receptor signaling pathway	0.009,441,203	7	1
Odontogenesis of dentin-containing tooth	0.010,274,693	8	1
Innate immune response	0.010,274,693	26	1
MRNA metabolic process	0.011,989,448	10	1
Toll-like receptor 3 signaling pathway	0.012,073,024	6	1
Enzyme regulator activity	0.012,073,024	28	1
Viral life cycle	0.012,215,227	7	1
Immune system process	0.012,215,227	47	1
Antigen processing and presentation of exogenous peptide antigen *via* MHC class II	0.012,228,456	8	1
Regulation of transcription, DNA-templated	0.012,489,378	93	1
Intrinsic apoptotic signaling pathway	0.013,169,422	6	1
Double-stranded DNA binding	0.013,169,422	11	1
*in utero* embryonic development	0.013,869,985	17	1
Intracellular transport of virus	0.016,044,445	3	1
Blood coagulation	0.016,044,445	16	1
Membrane organization	0.016,735,745	20	1
Entry into host cell	0.017,330,871	2	1
Transcription regulatory region DNA binding	0.018,173,983	16	1
Cellular response to potassium ion	0.020,863,254	3	1
Cytoplasmic stress granule	0.020,863,254	6	1
Cytoskeletal protein binding	0.027,511,888	25	1
Cell proliferation	0.029,665,962	24	1
Cellular component disassembly involved in execution phase of apoptosis	0.030,387,763	4	1
Methionine adenosyltransferase complex	0.038,551,825	2	1
RNA metabolic process	0.043,481,388	10	1
Positive regulation of macroautophagy	0.045,017,736	4	1

### 3.5 Identification of differentially expressed lncRNAs and circRNAs, and the construction of a ceRNA network

We screened the ENCORI and DIANA TOOLS databases and identified 11 lncRNAs that might target miRNAs (Figure 3C), including hsa-miR-147a/LINC00473; hsa-miR-212-5p/LIFR-AS1, EXTL3-AS1, KCNQ1OT1, and NEAT1; hsa-miR-668-3p/SNHG12, TTN-AS1, KCNQ1OT1, NEAT1, MEG3, and FTX; hsa-miR-942-5p/AC159540.1 and LINC01011. We also screened the ENCORI and CircBank databases and identified 6 circRNAs that may target miRNAs, including hsa-miR-147a/hsa_circ_0000175; hsa-miR-212-5p/hsa_circ_0004333, hsa_circ_0075961, hsa_circ_0001699, hsa_circ_0001731, and hsa_circ_0081673 (Figure 3D). Based on the miRNAs, lncRNAs, and circRNAs screened, a ceRNA network containing 4 miRNAs, 11 lncRNAs, and 6 circRNAs was constructed (Figure 3E). In addition, we selected LINC00473 in the ceRNA network to detect its expression; LINC00473 was highly expressed in placentas from preeclamptic women compared with those from normal pregnant women. This finding is consistent with the results of the microarray analysis (Figure 3F). The clinical information of the normal pregnant women and patients with PE is listed in Table 5.

**TABLE 5:** Clinical characteristics of samples for the validation of LINC00473.

Characteristics	NP (*n* = 10)	PE (*n* = 10)	*p*-value
Age (year)	34.40 ± 3.44	30.60 ± 3.53	0.025
Pre-pregnancy BMI	23.20 ± 2.38	28.13 ± 5.51	0.023
Gestation, (week)	38.84 ± 0.81	36.52 ± 3.75	0.085
Sbp (mmHg)	125.10 ± 9.37	161.60 ± 16.14	0.000
Dbp (mmHg)	72.30 ± 7.82	96.60 ± 10.09	0.000
Birth weight (g)	3,428.50 ± 428.31	2,673.00 ± 1,058.19	0.059

NP, normal pregnancy; PE, preeclampsia; BMI, body mass index; Sbp, Systolic blood pressure; Dbp, Diastolic blood pressure.

### 3.6 Construction of a PPI network

**FIGURE 4:**
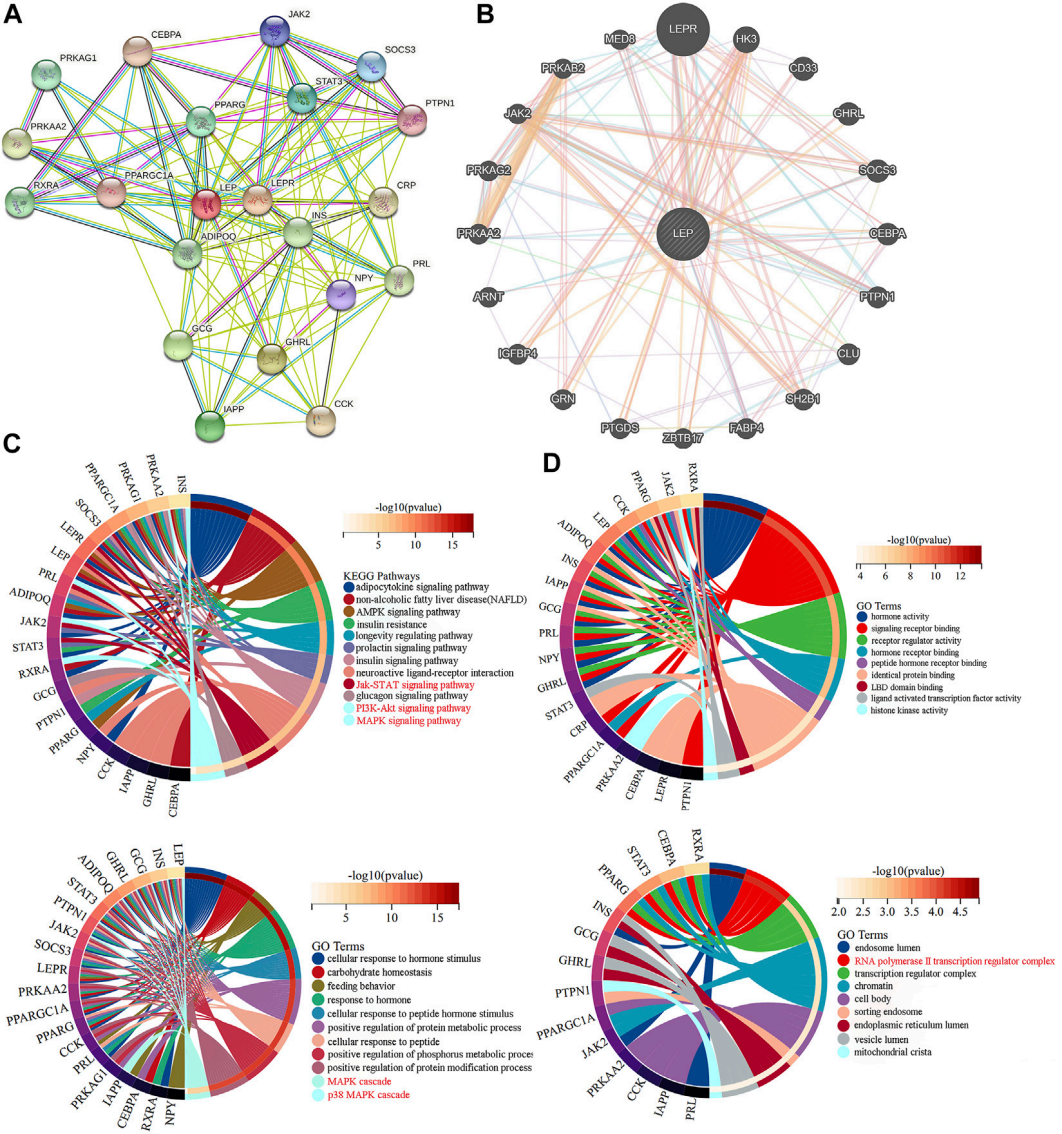
PPI networks exploring leptin-protein interactions. Leptin-binding proteins were identified using **(A)** the STRING tool, and **(B)** GeneMANIA. **(C,D)** KEGG and GO analyses of leptin-binding proteins from the STRING tool.

The STRING database analysis identified 20 leptin-binding proteins. As shown in Figure 4A, LEPR, GHRL, GCG, IAPP, PPARG, STAT3, SOCS3, JAK2, NPY, PTPN1, PPARGC1A, CEBPA, CCK, CRP, PRKAA2, PRL, INS, ADIPOQ, RXRA, and PRKAG1 were predicted to interact with leptin. Using GeneMANIA, we constructed a PPI network, which showed that LEPR, HK3, CD33, GHRL, SOCS3, CEBPA, PTPN1, CLU, SH2B1, FABP4, ZBTB17, PTGDS, GRN, IGFBP4, ARNT, PRKAA2, PRKAG2, JAK2, PRKAB2, and MED8 interact with leptin (Figure 4B). We then performed KEGG and GO analyses of the proteins predicted from the STRING database. KEGG analysis showed significant differences in the relevance of the PI3K-Akt and JAK/STAT signaling pathways. GO_BP classification revealed that proteins co-expressed with leptin were mainly involved in placental development, signal transduction, growth hormone signaling pathway *via* JAK/STAT, receptor signaling pathway *via* JAK/STAT, leptin-mediated signaling pathway, and female pregnancy. GO_CC annotations were mainly associated with the RNA polymerase II transcription regulator complex (Figures 4C,D). The protein KEGG analysis revealed that STAT3, PRL, SOCS3, LEPR, and JAK2 were associated with leptin in the JAK/STAT signaling pathway, suggesting that leptin may interact with these proteins to activate JAK/STAT signaling in PE. KEGG and GO analyses of leptin-binding proteins from geneMANIA are shown in Supplementary Figure S1.

### 3.7 RNA modification of LEP

**FIGURE 5:**
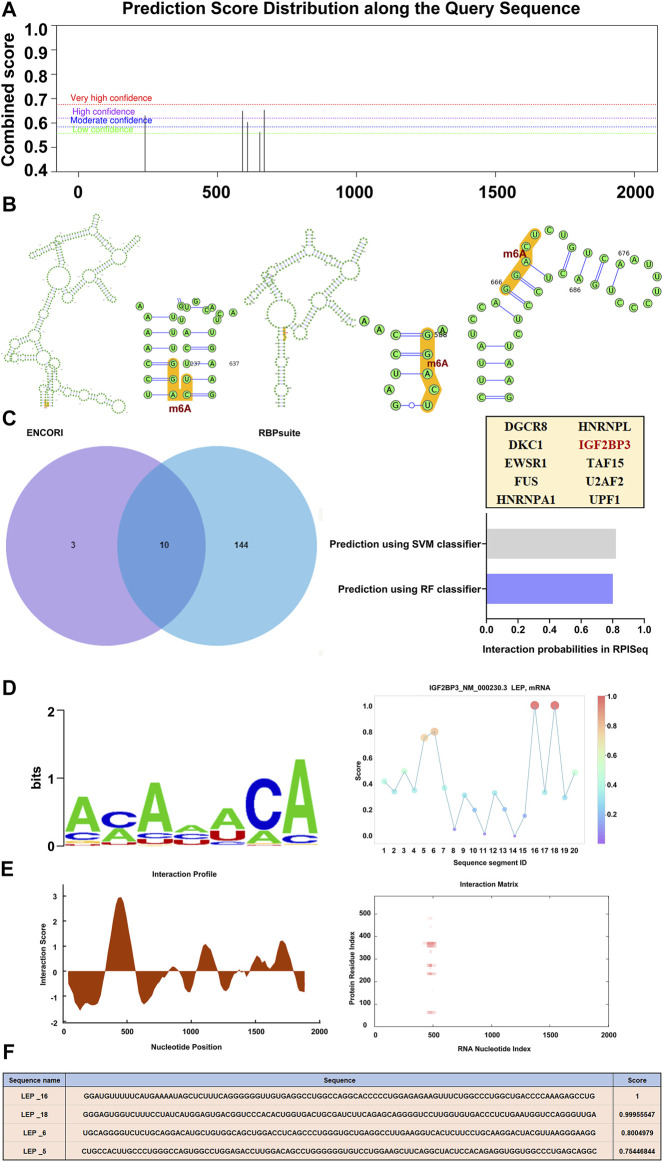
RNA modification of *LEP*. **(A,B)** The m6A modification site and its location in the RNA secondary structure (high confidence). **(C)** m6A-modified proteins that interact with *LEP* (IGF2BP3) were predicted using ENCORI and RBPsuite. The probability that IGF2BP3 interacts with *LEP* were predicted using RPISeq, which indicates RF classifier and SVM classifier scores. **(D,E)** The IGF2BP3 motif was analyzed using RBPsuite and catRPAID to screen for regions of the *LEP* mRNA most likely to be bound by IGF2BP3. **(F)** The binding site of IGF2BP3 on *LEP* was explored with RBPsuite.

Using SRAMP, a sequence-based m6A modification site predictor, we identified m6A modification sites in the *LEP* mRNA sequence, and displayed it on a high-confidence RNA secondary structure (Figures 5A,B). We then used ENCORI and RBPsuite to identify an m6A-modified protein that interacts with *LEP*, IGF2BP3. Using RPISeq, we predicted the probability of IGF2BP3 associating with *LEP*. Predictions with probabilities >0.5 are considered “positive,” indicating that the corresponding RNA and protein are likely to interact. For IGF2BP3, the RF classifier score was 0.8, and the SVM classifier score was 0.82 (Figure 5C). The IGF2BP3 motif was analyzed using the RBPsuite online database (Figure 5D). We used catRPAID to screen for *LEP* RNA regions that were most likely to be bound by IGF2BP3. The results revealed interaction profile peaks around the 500–600 nt site, indicating that IGF2BP3 may bind to *LEP* at this site (Figure 5E). We also used RBPsuite to explore the binding sites of IGF2BP3 on *LEP*, and the results indicated that IGF2BP3 may bind *LEP* at the 1,600–1,800 nt and 500–600 nt sites (Figure 5F).

### 3.8 Validation of LEP/leptin overexpression in clinical samples, the HTR-8/SVneo cell line, and a PE mouse model

**FIGURE 6:**
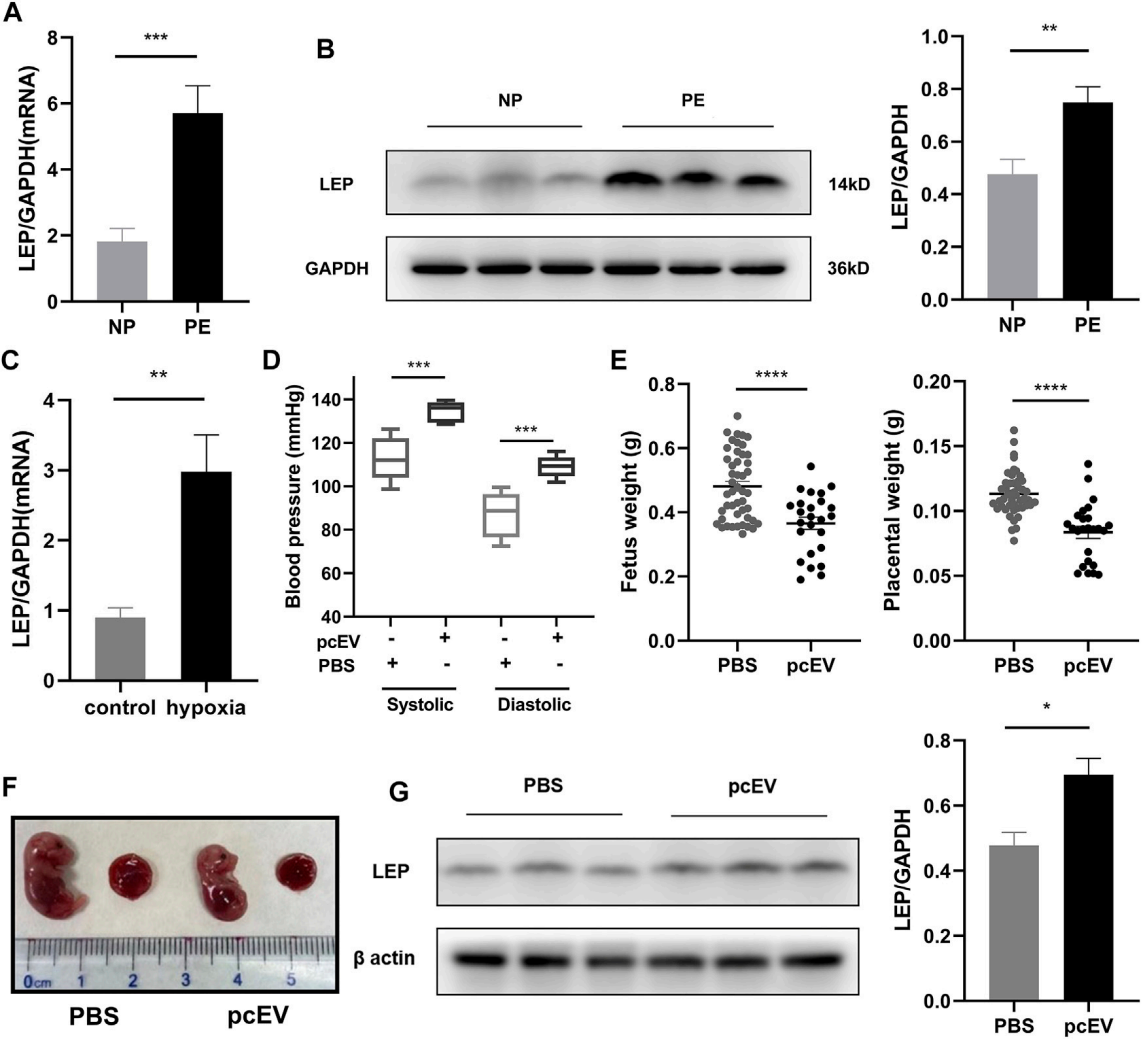
Validation of *LEP*/leptin expression in clinical samples, the HTR-8/SVneo cell line, and a PE mouse model. **(A)** RT-qPCR analysis of *LEP* expression in PE placental tissue samples (*n* = 22) compared with normal pregnancy placental tissue samples (*n* = 21). **(B)** Western blot analysis of leptin expression in PE placental tissue samples (*n* = 23) compared with normal pregnancy placental tissue samples (*n* = 20). **(C)** RT-qPCR analysis of *LEP* expression under normal oxygen concentration and hypoxia, control: normal oxygen concentration for 24 h, hypoxia: hypoxia condition for 24 h.**(D)** Blood pressure in PBS group (*n* = 5) compared with that in the pcEV group (*n* = 8). **(E)** Fetus weight and placental weight in PBS group (*n* = 47) compared with that in the pcEV group (*n* = 25). 5 litters in each group. **(F)** The appearance of fetus and placenta in PBS group compared with that in the pcEV group. **(G)** Western blot analysis of leptin expression in PBS group (*n* = 5) compared with that in the pcEV group (*n* = 8). * *p* < 0.05, ** *p* < 0.01, *** *p* < 0.001, **** *p* < 0.0001.

To further explore the expression of *LEP* in PE, we collected placental tissue samples and examined the expression of *LEP* in these specimens using RT-qPCR. Our results, shown in Figure 6A, reveal that *LEP* expression in PE placental tissue samples (n = 22) was significantly higher than its expression in normal pregnancy placental tissue samples (*n* = 21), which is consistent with the bioassay results. To confirm that *LEP* is upregulated at the protein level, western blotting was used to determine protein expression in placental tissue samples from normal pregnant women (*n* = 20) and PE patients (*n* = 23). The clinical information of the normal pregnant women and patients with PE is listed in Table 6. As shown in Figure 6B, leptin protein expression was significantly higher in the placental tissues of patients with PE than in normal pregnant women. In addition, the overexpression of *LEP* in PE was validated in the HTR-8/SVneo cell line and in a PE mouse model. In the hypoxia model of HTR-8/SVneo cell line, we found that *LEP* was upregulated at mRNA level under hypoxic conditions by RT-qRCR (Figure 6C). In the mouse model of PE, we found that the blood pressure (systolic and diastolic) in the PE group was significantly higher than that in the PBS group (Figure 6D), and the placental and fetal weights were significantly lower in the PE group than those in the PBS group (Figure 6E). It was also observed in appearance that the PE group was significantly smaller than the PBS group (Figure 6F). Importantly, our results indicate that *LEP* is overexpressed in the placentas of preeclamptic pregnant mice by western blot (Figure 6G).

**TABLE 6:** Clinical characteristics of samples for the validation of *LEP*.

Characteristics	NP (*n* = 21)	PE (*n* = 24)	*p*-value
Age (year)	33.57 ± 4.31	32.92 ± 3.91	0.596
Pre-pregnancy BMI	22.29 ± 2.74	27.48 ± 5.79	0.001
Gestation, (week)	38.84 ± 0.85	36.17 ± 3.26	0.001
Sbp (mmHg)	122.67 ± 8.96	158.96 ± 15.29	0.000
Dbp (mmHg)	74.33 ± 8.11	100.63 ± 11.72	0.000
Birth weight (g)	3,491.43 ± 566.37	2,713.26 ± 947.20	0.002

NP, Normal pregnancy; PE, Preeclampsia; BMI, Body mass index; Sbp, Systolic blood pressure; Dbp, Diastolic blood pressure.

## 4 Discussion

In this study, a screen of datasets in the GEO database revealed that *LEP* is significantly upregulated in PE. Using TargetScan, miWalk, miRDB, miRabel, ENCORI, CircBank, and other websites to identify *LEP*-targeting miRNAs, lncRNAs, and circRNAs, we constructed a lncRNA/circRNA-miRNA-*LEP* regulatory network that may regulate the abnormal expression of *LEP* in PE. We also performed single-gene KEGG, GO, and immune cell infiltration analyses, and by constructing a PPI network, we explored proteins interacting with *LEP* and their associated pathways and biological functions. We predicted m6A modification sites of *LEP* and related m6A-binding proteins, and importantly, we verified the overexpression of *LEP* mRNA and protein levels in clinical samples, the HTR-8/SVneo cell line and a PE mouse model using RT-qPCR and western blotting.

Leptin expression is higher in women experiencing normal pregnancies than in non-pregnant women, suggesting that leptin supports implantation and placental growth. Serum leptin levels increase weeks or months before the onset of PE, which may indicate that leptin itself is involved in the early-onset of PE. And studies have shown that serum leptin levels can be used as a biomarker to distinguish between early- and late-onset PE (Taylor et al., 2015; Hao et al., 2020; Liu et al., 2020). Leptin plays several roles in the regulation of pregnancy-related functions, while metabolic disorders and dynamic imbalances related to leptin during pregnancy play a decisive role in the occurrence and development of PE (de Knegt et al., 2021). Furthermore, using rat models, Ibrahim et al. proved that leptin can increase blood pressure and affect endothelial function, exhibiting pro-inflammatory properties (Ibrahim et al., 2013). In this study, we found that *LEP* was highly expressed in patients with PE, and we speculate that *LEP* may be a potential biomarker for the prevention, diagnosis, and treatment of PE. In the subsequent study, we can use siRNA or plasmid to knock down or over-express *LEP* in HTR-8/SVneo cell line, and experimentally verify whether it will change the migration and invasion ability of HTR-8/SVneo cells. At present, impaired trophoblast cell migration and invasion ability is considered to be one of the important mechanisms in the development of PE (Jiang et al., 2020). Thus, the role of *LEP* in the occurrence and development of preeclampsia was explored by targeting *LEP*.

The JAK/STAT pathway is a major intracellular signal transduction pathway. It is a critical downstream regulator of cytokines, hormones, and growth factors. Four members of the JAK family (JAK1, JAK2, JAK3, and TYK2) and seven members of the STAT family (STAT1-4, STAT5A/B, and STAT6) have been identified in mammals. Depending on the cytokine or growth factor that stimulates signaling, different combinations of JAKs and STATs are activated with a high degree of specificity (Dodington et al., 2018). At present, JAK/STAT signaling pathway has been studied in PE. Qu et al. found that hypoxia-inducible factor (HIF)-3α regulates the growth of EVT by up-regulating Fms-like tyrosine kinase receptor (Flt) 1/JAK/STAT signaling during hypoxia, which affects the progression of PE (Qu et al., 2021). However, there are few studies on the involvement of *LEP* in JAK/STAT signaling in PE. Previous studies have shown that leptin regulates placental amino acid transport by activating JAK/STAT signaling (JAK2 or STAT3) (Alijotas-Reig et al., 2017). STAT3 may play an important role in mediating trophoblast invasion (Zhang et al., 2018). Therefore, *LEP* may be involved in the development of PE through the JAK/STAT signaling pathway. In this study, the *LEP* single gene, miRNA, and PPI network-related gene KEGG analyses all showed that *LEP* might be associated with JAK/STAT signaling in PE. Therefore, we speculate that *LEP* may play an important role in the genesis and development of PE *via* this signaling pathway, which is a hypothesis worthy of further investigation.

Previous studies report that JAK/STAT signaling pathway has a certain relationship with immune infiltration. Wang et al. demonstrated that levamisole (LMS) inhibited T-cell activation and downregulated related molecules by inhibiting the activation of JAK/STAT signaling pathway (Wang et al., 2022). Zhou et al. found that the expansion of renal CD8+TRM cells may mediate and maintain renal inflammation and injury in lupus nephritis (LN), and the maintenance of renal CD8+TRM cell effector function depends on JAK/STAT signaling in LN kidneys (Zhou et al., 2020). All these studies have shown that JAK/STAT signaling pathway can regulate the occurrence and development of diseases by acting on immune cells through different signaling molecules. Therefore, we performed an immune cell infiltration assay in the hope that further studies can be conducted. An abnormal response of the maternal immune system to the placenta may be the first pathogenic step in PE, followed by a systemic inflammatory response involving the endothelium (Rambaldi et al., 2019). It is well known that not only does the number of macrophages change in patients with PE, but they also have a different state of polarization compared to patients with normal pregnancy. The total number of macrophages in the placenta of PE patients increased, while the number of M1 and M2 macrophages increased and decreased, respectively (Yao et al., 2019). Ji et al. showed that chemerin, by activating the CMKLR1/Akt/CEBPα axis, promotes the polarization of macrophages to an M1 subtype and inhibits the migration, invasion, and angiogenesis of trophoblast cells, thus participating in the initiation and development of PE (Ji et al., 2021). This finding is consistent with our results from the database screen. Our analysis of immune cell infiltration showed an increased percentage of M1 and a decreased percentage of M2 macrophages in PE patients compared to normal pregnant women. There were also differences in macrophages between the high and low *LEP* expression groups, suggesting that the level of *LEP* expression may be related to the change of macrophage infiltration in placenta. In our analysis of GSE75010, the expression of some other immune cells also changed. Our results suggest that the differences in immune cell infiltration in PE placentas may be related to differences in *LEP* expression.

The ceRNA networks constructed using bioinformatic tools are useful for exploring the role of mRNAs in disease. Recent studies have shown that lncRNAs and circRNAs can positively or negatively regulate miRNAs to influence the expression of downstream mRNAs and play an important role in the development of PE (Chen, 2016; Song et al., 2017). Zhang et al. constructed a lncRNA-related ceRNA network that regulates the expression of key genes in early-onset PE, including 21 lncRNAs, 3 mRNAs, and 69 miRNAs (Zhang et al., 2020). In their study, Yu et al. revealed that *SNHG16* expression is downregulated in PE placentas. *SNHG16* regulates trophoblast cell migration and invasion *via* the miR-218-5p/*LASP1* axis (Yu et al., 2021). Data from Ou et al. confirmed that hsa_circ_0111277 is upregulated in PE placenta, and that circ_0111,277 acted as a sponge for hsa-miR-494-3p in trophoblast cells by regulating the HTRA1/notch-1 signaling pathway, which inhibited the migration and invasion of these cells (Ou et al., 2020). However, there are still relatively few studies that have focused on the lncRNA/circRNA-miRNA-mRNA regulatory networks in PE. Therefore, we investigated lncRNA/circRNA-miRNA-*LEP* networks that may regulate *LEP* expression in PE, which is a likely target for developing new therapeutic strategies for PE. However, although our study describes a lncRNA/circRNA-miRNA-*LEP* regulatory network in PE, there is still a lack of studies on the pathological process of PE regulated by the lncRNA/circRNA-miRNA-*LEP* regulatory network, which may be a new challenge.

The m6A RNA modification is the most common internal modification in eukaryotic genes and plays a unique role in regulating mRNA metabolism, including mRNA splicing, output, localization, translation, and stability. M6A-modified mRNA also plays a vital role in many biological processes such as embryonic development, cell proliferation, and tumor formation (Zhou et al., 2021). Recently, studies on the m6A modification of RNA have become more extensive. Hou et al. found that LINC00460/DHX9/IGF2BP2 complex may regulate the expression of high mobility group AT-hook 1 (HMGA1) by recognizing the m6A modification site of HMGA1, thereby enhancing its mRNA stability and promoting the metastasis of colorectal cancer (Hou et al., 2021). Zhang et al. found that IGF2BP1 recognized and stabilized the mRNA of PEG10 in an m6A-dependent manner, enhancing the expression of PEG10, thereby accelerating the cell cycle and promoting EC progression (Zhang et al., 2021). Gu et al. found that increased *METTL3* expression and m6A RNA methylation were associated with increased *HNRNPC1/C2* expression in placental trophoblasts in PE, suggesting that abnormal m6A modification may be one of the causes of trophoblast cell dysfunction in PE (Gu et al., 2021). Guo et al. found that *ALKBH5* which is an m6A demethylase was significantly upregulated, and *PPARG* expression downregulated in PE placentas. *ALKBH5* interference reduced m6A levels on *PPARG*, increased the stability of *PPARG*, and promoted *PPARG* translation. Moreover, *ALKBH5* interference significantly promoted the proliferation, migration, and epithelial-to-mesenchymal transition of HTR-8/SVneo cells, as well as the inhibition of apoptosis and oxidative stress (Guo et al., 2022). Wang et al. found that *HSPA1A* may be involved in the pathophysiology of PE, and showed that m6A modification significantly upregulated the expression of *HSPA1A* and its protein, suggesting that m6A plays a key role in gene expression regulation and is involved in the pathophysiological processes underpinning PE (Wang et al., 2020). Furthermore, studies have indicated that m6A may play an important role in blood pressure regulation (Mo et al., 2019). Based on numerous studies of m6A in PE and other diseases, we sought to explore whether m6A affects the translational stability of *LEP* in PE. Currently, few studies which have focused on the RNA modification of *LEP* in PE, and our study found that there is an m6A modification site on *LEP*, as well as an m6A binding protein, IGF2BP3, that may interact with *LEP*. RNA modification often relies on consensus motifs to form secondary structures that bind to RNA-modifying proteins known as writers, readers, and erasers. Therefore, the IGF2BP3 motif was analyzed. In addition, we found that IGF2BP3 is likely to bind *LEP* at the 500–600 nt site. Interestingly, the effect of m6A modification on targeted mRNAs depends primarily on the different m6A binding proteins. A study by Wang et al. revealed that IGF2BP3 affects the stability of TMBIM6 by participating in the m6A modification of TMBIM6 (Wang et al., 2021). Therefore, our study suggests that IGF2BP3 may bind *LEP* mRNAs to influence the development of PE. We hypothesize that RNA modification of *LEP* might stabilize the transcript and promote the expression of leptin.

Notably, the expression of *LEP*/leptin, and LINC00473 in clinical PE tissues were detected by both RT-qPCR and western blot analyses. We confirmed that the expression of *LEP*/leptin, LINC00473 in PE was significantly higher than that in normal pregnancy. Together, these data confirmed that overexpression *LEP*/leptin may indeed play a role in PE. In addition, the high expression of *LEP* in PE was verified in both the hypoxic HTR-8/SVneo cell line and the PE mouse model. By investigating the expression of *LEP* in PE *in vitro* and *in vivo*, we further found that *LEP* may play a role in the pathogenesis of PE. The study by Han et al. (2020) successfully generated a mouse model of PE. Injection of pcEVs into pregnant C57BL/6J mice to increase circulating pcEV levels leads to preeclampsia-like changes such as hypertension, proteinuria, and other pathological changes such as vascular injury and constriction. Our results also showed that pregnant C57BL/6J female mice with increased circulating pcEVs by pcEVs injection developed hypertension and fetal growth restriction. These provided a basis for us to validate the high expression of *LEP* in PE using this PE mouse model. However, this study does have some limitations. Although our investigations involved related pathway analyses, we didn’t conduct in-depth research or further study on the predicted miRNAs, lncRNAs, and circRNAs. Further *in vitro* and *in vivo* experiments are needed to explore whether the upstream predicted lncRNAs or circRNAs binds to miRNAs and whether miRNAs binds to *LEP*, and then to investigate the regulatory effect of upstream non-coding RNAs (ncRNAs) on *LEP* in the case of knockdown or overexpression, and its effect on the occurrence and development of PE. It would be worthwhile exploring these regulatory RNAs in future studies to better understand the etiology and pathological mechanisms of PE. In addition, validation of *LEP* as a possible biomarker for PE by using clinical samples collected in the third trimester with a clear diagnosis of normal pregnancy or PE at the time of collection has certain limitations. This requires us to conduct prospective studies, such as examining the levels of *LEP* in peripheral blood during pregnancy, to predict PE.

## 5 Conclusion

In this study, database screening identified the *LEP* gene to be upregulated in PE and bioinformatics tools were used to predict the corresponding miRNAs, lncRNAs, and circRNAs, and construct a lncRNA/circRNA-miRNA-*LEP* regulatory network. We then investigated the function of *LEP* by KEGG, GO, and immune cell infiltration analyses, in addition to predicting m6A modification sites and corresponding binding proteins. Finally, we verified the high expression of *LEP* in clinical samples, the hypoxic HTR-8/SVneo cell line and the PE mouse model at an mRNA and protein level. These data lay the foundation for further research on the role of leptin in the pathogenesis of PE, which could lead to a better theoretical basis for predicting, preventing, and treating PE in clinical settings.

## Data Availability

The datasets presented in this study can be found in online repositories. The names of the repository/repositories and accession number(s) can be found in the article/Supplementary Material.
