# Supplementary material for: Bioinformatics analysis combined with clinical sample screening reveals that leptin may be a biomarker of preeclampsia
Source: Front Physiol. 2023 Jan 4;13:1031950. doi: 10.3389/fphys.2022.1031950 (PMC9846503; doi:10.3389/fphys.2022.1031950)
Supplement: Supplementary file 1 [file DataSheet1.docx]

**Supplementary material**


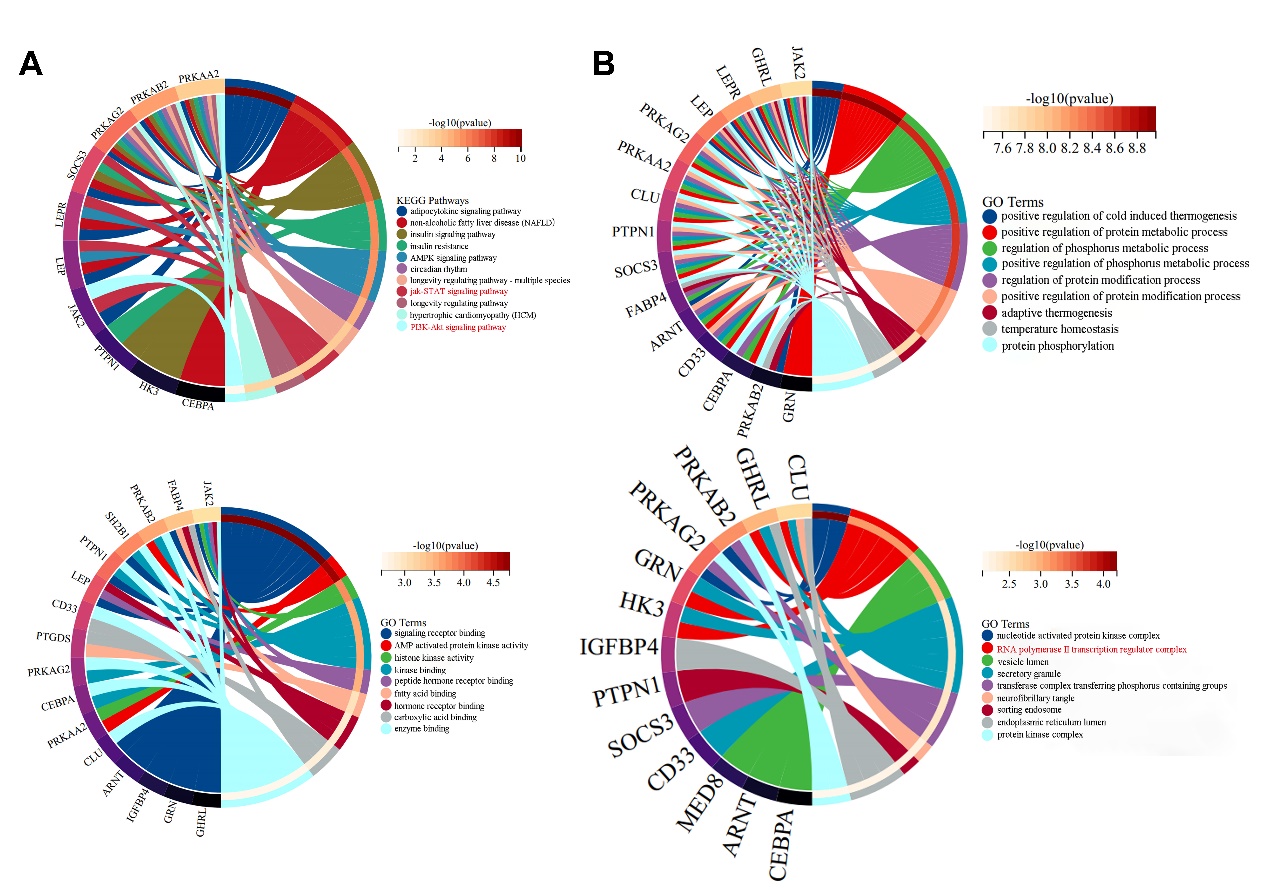


**Supplementary figure 1 (**A) KEGG and (B) GO analyses of leptin-binding proteins from geneMANIA


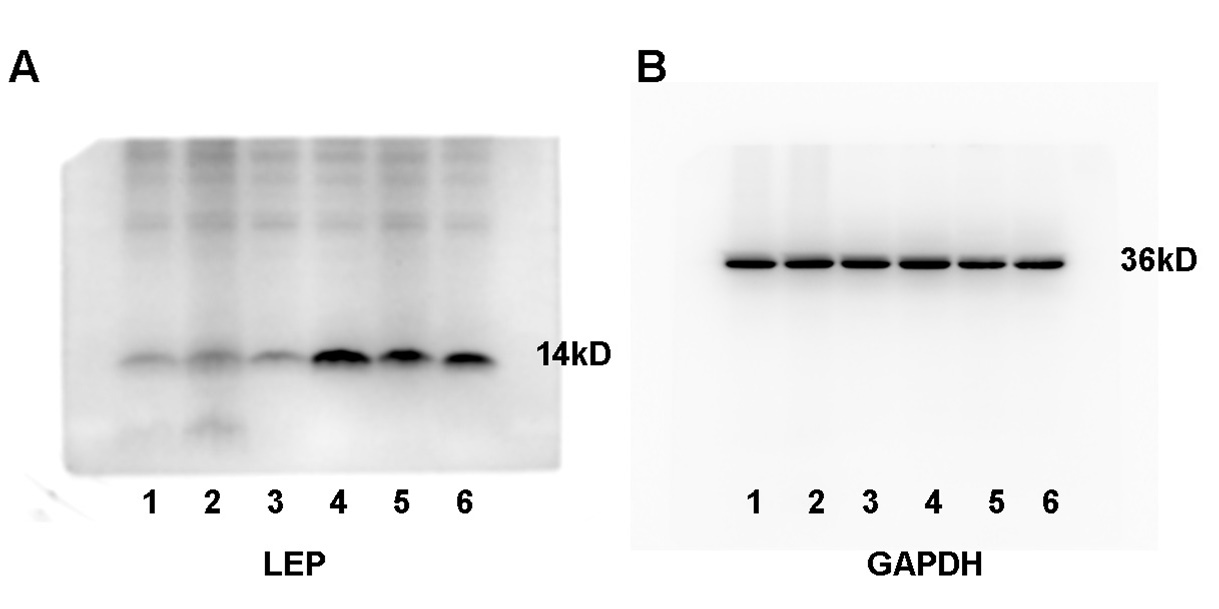


**Supplementary figure 2** the whole uncropped images of the original western blotting

1. LEP, its molecular weight is 14kD. (B) GAPDH, its molecular weight is 36kD.

(lanes 1, 2, 3: Placental villus tissue of normal pregnancy; lanes 4, 5, 6: Placental villus tissue of PE).
